# A population-based comparison of treatment, resource utilization, and costs by cancer stage for Ontario patients with HER2-positive breast cancer

**DOI:** 10.1007/s10549-020-05976-w

**Published:** 2020-10-22

**Authors:** Christine Brezden-Masley, Kelly E. Fathers, Megan E. Coombes, Behin Pourmirza, Cloris Xue, Katarzyna J. Jerzak

**Affiliations:** 1 Division of Medical Oncology and Hematology, Faculty of Medicine, Mount Sinai Hospital, University of Toronto, Toronto, ON Canada; 2 Department of Medical Affairs, Hoffmann-La Roche Limited, Mississauga, ON Canada; 3 Market Access and Pricing Department, Hoffmann-La Roche Limited, Mississauga, ON Canada; 4 Division of Medical Oncology and Hematology, Faculty of Medicine, Sunnybrook Odette Cancer Center, University of Toronto, Toronto, ON Canada

**Keywords:** Breast neoplasms, Drug therapy, Epidemiologic studies, Health expenditures, Health services research, Receptor ErbB-2

## Abstract

**Purpose:**

We sought to expand the currently limited, Canadian, population-based data on the characteristics, treatment pathways, and health care costs according to stage in patients with human epidermal growth factor receptor-2 positive (HER2+) breast cancer (BC).

**Methods:**

We extracted data from the publicly funded health care system in Ontario. Baseline characteristics, treatment patterns, and health care costs were descriptively compared by cancer stage (I–III vs. IV) for adult women diagnosed with invasive HER2+ BC between 2012 and 2016. Resource use was multiplied by unit costs for publicly funded health care services to calculate costs.

**Results:**

Overall, 4535 patients with stage I–III and 354 with stage IV HER2+ BC were identified. Most patients with stage I–III disease were treated with surgery (4372, 96.4%), with the majority having a lumpectomy, and 3521 (77.6%) received radiation. Neoadjuvant (NAT) and adjuvant (AT) systemic treatment rates were 20.1% (*n* = 920) and 88.8% (*n* = 3065), respectively. Systemic treatment was received by 311 patients (87.9%) with metastatic HER2+ BC, 264 of whom (84.9%) received trastuzumab. Annual health care costs per patient were nearly 3 times higher for stage IV vs. stage I–III HER2+ BC.

**Conclusion:**

Per-patient annual costs were substantially higher for women with metastatic HER2+ BC, despite less frequent exposure to surgery and radiation compared to those with early stage disease. Increasing NAT rates in early stage disease represent a critical opportunity to prevent recurrence and reduce the costs associated with treating metastatic HER2+ BC.

**Electronic supplementary material:**

The online version of this article (10.1007/s10549-020-05976-w) contains supplementary material, which is available to authorized users.

## Introduction

Overall, human epidermal growth factor receptor-2 (HER2) positivity is seen in approximately 15–20% of breast carcinomas [1]. HER2-targeted therapy has revolutionized the treatment of HER2+ breast cancer (BC), and its use in the curative setting has changed the natural course of the disease, achieving outcomes similar to those of patients with HER2-negative disease for a historically poor prognosis subpopulation [2]. In the metastatic setting, chronic management has become a clinical reality, with trial and epidemiological data proving that HER2-targeted therapy prolongs survival [2, 3].

Trastuzumab is the earliest and most extensively studied HER2-targeted therapy in BC, and its efficacy has been demonstrated in both early and metastatic disease [2, 4]. Additional HER2-targeted therapies have since been approved including lapatinib, pertuzumab, trastuzumab emtansine (TDM1), and neratinib. In patients with HER2+ breast cancer that also express hormone receptors (HR+), standard HER2-targeted therapy with chemotherapy remains the most common approach upfront, with substitution of chemotherapy for endocrine therapy in the maintenance phase of treatment [5].

Canadian population-based studies focused on HER2+ BC are sparse and the majority address only clinical characteristics and outcomes, typically in early or metastatic BC, without comparison between stages. There is also very little information available regarding differential resource utilization in populations of patients with HER2+ BC. We therefore sought to assess the occurrence, management, resource utilization, and cost by cancer stage of Ontario residents with HER2+ BC.

## Methods

### Study design

We conducted a retrospective, observational, population-based study to assess the treatment, resource utilization, and public health care costs for a cohort of Ontario women diagnosed with stage I–III versus stage IV HER2+ BC. The study was approved by the Ontario Cancer Research Ethics Board in 2017 and conducted in 2019 by ICES using all relevant databases under their purview.

ICES is an independent non-profit organization that houses de-identified population-based health and social data on publicly funded services provided in Ontario. Cases are linked across databases by their unique Ontario Health Insurance Plan (OHIP) number. In Ontario, all Canadian citizens and permanent residents are eligible to receive publicly funded hospital care, most physician services, outpatient and emergency services, and, for those 65 years of age or older or on social assistance, prescription drug coverage. Supplemental drug funding is also provided by the government through special programs within the Ontario Drug Benefit (ODB) program or the New Drug Funding Program (NDFP).

Incident cases of invasive BC [6] in adult women (18–105 years old) pathologically diagnosed between Apr 1, 2012 and Mar 31, 2016 were extracted from the Ontario Cancer Registry (OCR). This timeframe was selected based on feasibility data from ICES and took into consideration availability of laboratory test results for HER2 and HR status as well as the target population of 40,000 women with breast cancer. Those diagnosed with a secondary non-BC malignancy were excluded from the analysis, as were those with incomplete/invalid records (i.e., missing age/gender) (Fig. S1). The final cohort for this substudy only included patients with a known and positive HER2 test result, known HR status and a documented cancer stage. Molecular subtype was determined from synoptic pathology reports. HER2 positivity and HR status were defined according to guidelines relevant locally at the time [1, 7, 8]. Patients were assigned to the stage I–III or stage IV cohort based on their stage at initial diagnosis; therefore, all patients in the stage IV cohort had de novo metastatic disease. Patients were followed until the earliest of the following: date of last contact with the health care system, end of OHIP eligibility, death, or end of study, which was Mar 31, 2017.

### Measures and data sources

Variables of interest for data collection included age, rurality [9], comorbidity index [10], income status [11], and various tumor characteristics. American Joint Committee on Cancer (AJCC) disease stage at diagnosis was reported according to the Collaborative Staging Methodology (v. 1.0, 2004) which incorporates TMN information [12]. Tumor characteristics of interest included histologic grade (reported according to the Nottingham combined scoring system) [13], laterality, pathologic tumor size, and lymph node status.

Treatments received (surgery, radiation, and/or systemic therapy), time between diagnosis and start of each treatment modality, and the frequency of targeted or endocrine therapy use were gathered. The authors reviewed database treatment codes and ensured queries related to systemic therapy were specific to anti-cancer therapies. Surgery dates and types were derived from the Canadian Institute for Health Information (CIHI) Discharge Abstract and Same-Day Surgery databases. Rates of radiation therapy (RT) were calculated using radiation exposure data captured in the OHIP, National Ambulatory Care Reporting System (NACRS), and/or Activity Level Reporting (ALR) databases between diagnosis and study end. Rates of systemic therapy were calculated using drug exposure data from the OHIP, NACRS, ALR, NDFP, and/or ODB databases.

For patients with stage I–III disease, systemic therapy was categorized as neoadjuvant (NAT, occurring before surgery) or adjuvant (AT, occurring within 24 weeks of surgery—a broad window intended to ensure the capture of systemic therapy in case of delay following locoregional therapy). For patients with stage IV disease, first and second lines were defined as the first or second therapy, respectively, following metastatic diagnosis. Since the majority of systemic anti-cancer therapies are reimbursed (by ODB or NDFP), dispensed, and administered by the cancer clinics, the ALR database was considered the most comprehensive provincial record of cancer regimens received.

Health resource utilization measures included number of events/uses as well as length of stay where applicable and were queried in the databases outlined in Table S1. Costs were determined by multiplying the health resource utilized by the unit cost. Unit costs for emergency room visits, day hospitalizations/surgeries, and inpatient/rehabilitation stays were sourced from CIHI and the Ontario Case Costing Initiative. Costs for biopsies, imaging, physician visits, and laboratory tests were sourced from the Ontario Ministry of Health and Long-Term Care (MOHLTC). Health service cost components were summed to calculate the total direct cost for the full period of care. To estimate annual direct health care costs per patient, total costs over the study period were divided by the period of care and the number of patients. All reported costs were inflated to 2017 Canadian dollars using the Consumer Price Index calculator [14].

For some reporting, costs were combined into themes, as follows:

- Continuous care = long-term care + complex continuing care.
- Pharmaceutical (drug only) = ODB + NDFP.
- Inpatient = hospital + mental health + rehabilitation.
- Ambulatory non-cancer = emergency department + dialysis clinic visits.

Hospital outpatient cost data were derived from the MOHLTC and defined as billings involving day surgery, medical day care, or clinic care related to clinic attendance, rehabilitation services, or diagnostic tests. These costs were then linked to OHIP records using a validated algorithm [15].

### Statistical methods

Considering the descriptive nature of our study and that there were approximately 9614 new cases of BC each year in Ontario [16], our sample size was fixed as the number of cases identified over the four-year period of the study.

Results are reported using descriptive statistics for center (mean and median) and dispersion (SD and interquartile range [IQR], respectively) for all continuous variables. Categorical variables were summarized using counts and percentages. In accordance with ICES policies, cells with fewer than six patients and any interrelated cells were suppressed to prevent re-identification.

## Results

### Patient characteristics

Among the 34,340 women newly diagnosed with BC and meeting the criteria for inclusion, 4902 (14.3%) had HER2+ BC and known HR status. Notably, 3914 patients had an unknown histologic subtype, and 13 had no reported disease stage and were therefore excluded from further analyses.

The mean age of women with stage I–III HER2+ BC was 57.7 years (± 13.4) and 58.9 (± 14.5) in those with stage IV disease. Table 1 highlights key demographic and tumor characteristics observed in the cohort of patients with staged disease (*n* = 4889) and compares subcohorts with stage I, II, or III versus stage IV disease at diagnosis. Nearly two thirds of patients were under the age of 65. Patients were fairly evenly distributed among index year and income quintiles. Comorbidity data were missing for a significant proportion of patients. Approximately 62% of patients with stage I–III and 78% with stage IV HER2+ BC were found to have tumors 2 cm or larger and just over half of tumors staged I–III were poorly differentiated (grade 3). Patients with stage IV disease had a higher frequency of HR negative pathology.

**Table 1:** Baseline characteristics of Ontario patients diagnosed with HER2+ 2+ breast cancer (*n* = 4889) by stage at diagnosis (2012–2016)

	Stage I-III (*n* = 4535)		Stage IV (*n* = 354)	
	No.	%	No.	%
Age, years				
18–64	3162	67.9	225	63.6
65+	1373	30.3	129	36.4
Index fiscal year				
2012	1044	23.0	89	25.1
2013	1115	24.6	80	22.6
2014	1187	26.2	81	22.9
2015	1189	26.2	104	29.4
Rurality of residence				
Missing	6	0.1	0	0.0
No	4020	88.6	308	87.0
Yes	509	11.2	46	13.0
Income quintile				
Missing	17^a^	0.4^a^	3^a^	0.8^a^
1-Lowest	736^a^	16.2^a^	59^a^	16.7^a^
2	864	19.1	76	21.5
3	905	20.0	62	17.5
4	1020	22.5	78	22.0
5-Highest	993	21.9	76	21.5
Charlson comorbidity index				
Missing	3242	71.5	269	76.0
Mean ± SD	0.59 ± 1.18		0.91 ± 1.68	
Tumor size				
No mass found	10^a^	0.2^a^	3^a^	0.8^a^
<1 cm	537^a^	11.8^a^	3^a^	0.8^a^
1 to < 2 cm	1117	24.6	16	4.5
2 to < 5 cm	2163	47.7	136	38.4
5 cm or greater	658	14.5	139	39.3
Other^b^	10	0.2	7	2.0
Unknown	39	0.9	51	14.4
Laterality of the primary				
Right	2268	50.0	177	50.0
Left	2267	50.0	174^a^	49.2^a^
Paired site	0	0.0	3^a^	0.8^a^
Lymph node status				
Negative	1924	42.4	160	45.2
Positive	2423	53.4	17	4.8
Unknown	188	4.1	177	50.0
Tumor grade				
Grade 1	124	2.7	6^c^	1.7^c^
Grade 2	1338	29.5	61	17.2
Grade 3	2377	52.4	99	28.0
No exam/unknown	696	15.3	191^a^	54.0^a^
Disease stage				
I	1412	31.1	0	0.0
II	2107	46.5	0	0.0
III	1016	22.4	0	0.0
IV	0	0.0	354	100.0
HR status				
ER−/PR−	1453	32.0	142	40.1
ER+/PR+	2240	49.4	128	36.2
ER+/PR−	773	17.0	76	21.5
ER−/PR+	69	1.5	8	2.3

*ER* estrogen receptor, *HR* hormone receptor, *PR* progesterone receptor

^a^Mid-point of suppressed data range; *n* = ±2

^b^Diffuse disease or Paget’s disease of the nipple with no tumor

^c^Mid-point of suppressed data range; *n* = ±4

Of the 4889 patients included in the analysis, 488 (10.0%) died within the timeframe of study follow-up (median 33 months [IQR: 22–46]). This included 318 patients with stage I–III (7.0%) and 170 (48.0%) with stage IV disease with a median follow-up of 34 (IQR: 22–47) and 21 (IQR: 11–35) months, respectively.

### Treatment

Rates of surgery and RT were lower in the stage IV cohort compared with the stage I–III cohort, while systemic treatment rates were similar (Table 2).

**Table 2:** Treatment received by Ontario patients diagnosed with HER2+ breast cancer (*n* = 4889), by stage at diagnosis (2012–2016)

Treatment modality	Stage I-III (n = 4535)		Stage IV (n = 354)	
	No.	%	No.	%
Surgery (within 1 year of diagnosis)	4372	96.4	82	23.2
No surgery	163	3.6	272	76.8
Lumpectomy^a^	2582	56.9	31	8.8
Mastectomy^a^	1,744^b^	38.5^b^	49^b^	13.8^b^
Lumpectomy followed by mastectomy^a,c^	31^b^	0.7^b^	3^b^	0.9^b^
Lymph node excision only	11^b^	0.2^b^	3^b^	0.9^b^
Systemic therapy	4113	90.7	311	87.9
Trastuzumab	3570	78.7	264	74.6
Pertuzumab	166	3.7	185	52.3
Trastuzumab emtansine	54	1.2	64	18.1
Lapatinib	10	0.2	16	4.5
Endocrine therapy	2270	50.1	102	28.8
Aromatase inhibitors	1332	29.4	67	18.9
Radiation therapy	3521	77.6	213	60.2

^a^With or without lymph node excision

^b^Mid-point of suppressed data range; *n* = ±2

^c^Entry occurs on same surgery record

In the group of patients with stage I–III disease receiving surgery (*n* = 4372, 96.4%), the mean number of surgeries was 1.17 (± 0.40) and the median number of days between diagnosis and first surgery was 38 (IQR: 25–70) (Table S2). Among patients treated with upfront surgery for early stage disease (*n* = 3452, 79.0%), 3065 (88.8%) received AT. The remaining 920 surgical patients with stage I–III disease (21.0%) received NAT starting a median of 29 days (IQR: 21–40) after diagnosis and underwent surgery a median of 146 days (IQR: 133–167) after the start of NAT. Approximately 98.5% (*n* = 906) of patients who received NAT went on to receive AT after surgery. AT was started within a median of 42 days (IQR: 26–55) of surgery. Of the patients undergoing surgery, 214 (4.9%) received neither systemic nor RT. Trastuzumab was the most frequently used targeted therapy in patients with stage I–III HER2+ BC (*n* = 3570, 78.7%), and endocrine treatment was received by 2270 (50.1%) (Table 2).

For patients diagnosed with stage IV HER2+ BC, surgery was the least common treatment modality (*n* = 82, 23.2%) compared with RT (*n* = 213, 60.2%) and systemic therapy (*n* = 311, 87.9%) (Table 2). Of those not undergoing surgery (*n* = 272), 235 (86.4%) received systemic treatment a median of 36 days (IQR: 22–51) after diagnosis, and 160 (58.8%) received RT. Further data on treatments received in patients with stage I–III and IV HER2+ 2+ BC can be found in Tables S2 and S3, respectively. Trastuzumab was the most frequently used targeted therapy (*n* = 264, 74.6%) for metastatic disease while endocrine therapy was received by 102 patients (28.8%) (Table 2).

### Resource utilization and costs

**Fig. 1:**
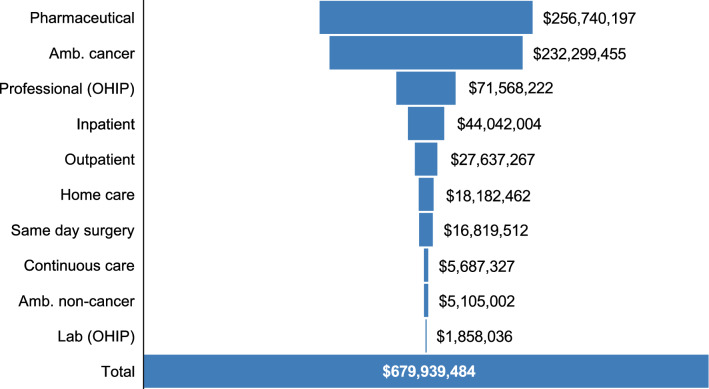
Total costs by resource type for Ontario patients with HER2+ breast cancer (*n* = 4889), irrespective of stage, across study period (2012–2017). Abbreviations: *Amb.* ambulatory, *OHIP* Ontario Health Insurance Plan

The full HER2+ cohort was responsible for a total measured resource consumption of $679,939,484 between 2012 and 2017. Pharmaceuticals and cancer clinic visits combined accounted for over half of the costs incurred (38% and 34%, respectively) (Fig. 1).

For patients with stage I–III HER2+ BC, the average annual per-patient total cost was $54,852, compared with $159,348 for patients with stage IV disease (Tables 3 and S4). Average annual per-patient costs were higher for patients with stage IV HER2+ BC for all resource use categories except same-day surgery, which was higher for patients with stage I–III disease. Pharmaceuticals, outpatient cancer clinic visits, and OHIP professional fees were the most costly resources used in the stage I–III cohort, whereas pharmaceuticals, inpatient hospital services, and outpatient cancer clinic visits were the primary contributors to annual costs in the stage IV cohort.

**Table 3:** Average annual per-patient cost by resource for patients with HER2+ breast cancer (*n* = 4889), by stage at diagnosis (Ontario, 2012–2017)

	Stage I-III (*n* = 4535)		Stage IV (*n* = 354)	
	Cost	% Of total cost	Cost	% Of total cost
Total	$54,852	100%	$159,348	100
Pharmaceutical	$19,454	36	$51,700	32
Ambulatory cancer	$19,146	35	$28,376	18
Professional (OHIP)	$5922	11	$15,687	10
Inpatient	$3942	7	$44,447	28
Outpatient	$2238	4	$5876	4
Home care	$1499	3	$6146	4
Same-day surgery	$1468	3	$483	0
Continuous care	$572	1	$4841	3
Ambulatory non-cancer	$466	1	$1612	1
Lab (OHIP)	$145	0	$180	0

*OHIP* Ontario Health Insurance Plan

**Fig. 2:**
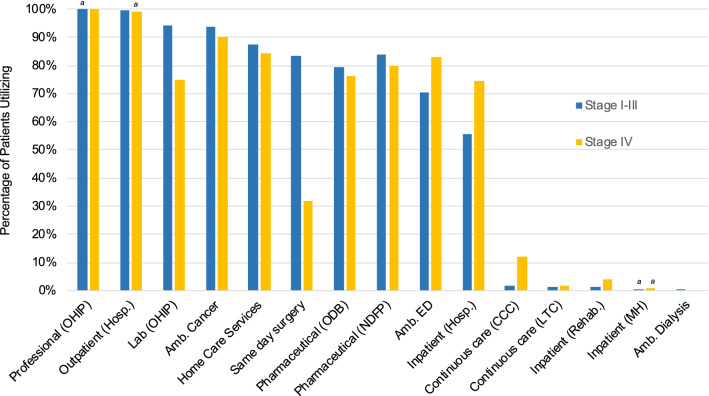
Proportion of patients with HER2+ breast cancer, by stage, utilizing each health care resource (Ontario, 2012–2017). ^*a*^mid-point of suppressed data range; *n* = ±2. Abbreviations: *Amb.* ambulatory, *CCC* complex continuing care, *ED* emergency, *Hosp.* hospital, *LTC* long-term care, *MH* mental health, *NDFP* New Drug Funding Program, *ODB* Ontario Drug Benefit, *OHIP* Ontario Health Insurance Plan, *Rehab.* rehabilitation

OHIP professional services, hospital outpatient services, outpatient cancer clinics, home care services, and drug funding programs were highly utilized by a similar proportion of patients regardless of disease stage (Fig. 2 and Table S4). Proportionally fewer patients with de novo stage IV disease required OHIP lab services or same-day surgery services compared to patients with stage I–III BC. Emergency, inpatient, and complex continuing care were, in contrast, utilized by a higher proportion of patients with stage IV HER2+ BC.

Of the 4889 patients with HER2+ BC, 2777 (57%) had at least one inpatient hospital stay. Among them, patients with stage IV disease had an average number of visits six times that of those with stage I–III disease and a substantially longer average length of stay (46.0 versus 4.4 days, respectively). Average annual outpatient services were twice as costly, while homecare services were four times as expensive for patients with stage IV versus stage I–III HER2+ BC. Annual costs related to emergency hospital services, pharmaceutical utilization, and complex continuing care were approximately tripled in the stage IV versus the stage I–III cohort using these resources (Table S4).

## Discussion

### Patient characteristics

In our Ontario BC cohort, 14.3% of women diagnosed between 2012 and 2016 were categorized as having HER2+ breast cancer, in line with expected rates (15–20%) [1] and recent large population-based reports [17]. Importantly, an Ontario review of HER2+ classifications found no change in the proportion of BCs considered HER2+, despite a 2013 change in guidelines for HER2 testing [18].

Overall, 7.2% of patients with HER2+ BC in our study were diagnosed with de novo stage IV disease. This is slightly higher than the rate of all stage IV cancers in Ontario (< 5%) [19] and underscores the findings of a US study that hypothesized that an increasing incidence of stage IV tumors, despite increased screening, is likely due to the growing contribution of aggressive phenotypes like triple negative and HER2+ BC [20]. In a recent Ontario-based study regarding BC screening patterns, triple negative and HER2+ BCs were diagnosed at a more advanced stage than HR+/HER2- tumors, possibly owing to faster growth rates rather than a relative inability to detect them in screening [21]. This aligns with our observation that 77.7% of patients with stage IV HER2+ BC in our cohort had primary tumors that were larger than 2 cm.

In our study, women diagnosed with metastatic HER2+ BC more commonly had HR− (40.1%) tumors compared to women diagnosed with stage I–III disease (32.0%). In general, the proportion of patients in each stage, grade, lymph node, and HR status were similar to those reported in other large real-world datasets [17, 22–28]. Unfortunately, a higher proportion of patients in our cohort had larger tumors at diagnosis (62.1% ≥ 2 cm in stage I–III HER2+ tumors) compared with an earlier Canadian [23] and a large US population-based study [17] (51.3% and 47.7% ≥ 2 cm, respectively).

### Treatment

Surgery was a mainstay of treatment for Ontario stage I–III HER2+ BC patients with an impressive 96% undergoing either lumpectomy (> 58%) and/or mastectomy (> 39%). This is encouraging given the proven survival benefits of surgery and is higher than [24] or in line with [22, 23, 29, 30] those reported in other cohorts. Nearly 78% of patients with stage I–III HER2+ BC in our study received RT, a rate that is similar to or higher than other population-based reports [22, 23, 28, 31].

Interestingly, around 62% of women with stage I–III HER2+ BC in our study had tumors > 2 cm, and 53.4% were lymph node positive, yet only 21% of patients undergoing surgery received NAT. This may in part be attributable to a lack of core biopsy testing during the period of our study, which is still an unfortunate clinical reality for many Ontario centers. In the absence of upfront pathological results, patients with HER2+ BC may miss the opportunity for targeted NAT, early disease control, and tumor downstaging, which may allow for less extensive breast surgery. Furthermore, the evaluation of pathologic complete response after NAT is of benefit in terms of prognostication [32] and determines eligibility for TDM1, since this agent was recently proven to be effective among patients with residual invasive disease post-NAT [33].

In our cohort, nearly 87% of women with stage I–III HER2+ BC had tumors > 1 cm, which, at the time, meant they were eligible to receive (neo)adjuvant trastuzumab for 1 year [34]. However, only 79% of the early HER2+ BC cohort received trastuzumab. Although this rate of exposure is in line with other contemporary real-world reports [23, 35], reasons for non-exposure should be explored further. Importantly, during our study, trastuzumab was not publicly funded for breast tumors < 1 cm (~ 12% of our stage I–III cohort). In addition, our study window predated evidence supporting novel approaches to NAT, such as dual blockade with trastuzumab–pertuzumab in HER2+ disease [36].

Despite funding starting only part way through our study [37], pertuzumab was received by half of all patients with metastatic HER2+ BC.

### Resource utilization and costs

DaCosta Byfield et al. [28] reported a mean 12-month per-patient cost to be $176,779 USD in the first year of treatment for early HER2+ BC based on data from a commercially insured US population. Our yearly costs in patients with early HER2+ BC were lower than in the US study but were averaged for the duration of follow-up, as opposed to a more treatment-intense first year alone (as in the US study); further, both the patient population and health care system differ greatly in Canada compared to the US.

Considering methodological differences and variability in health care systems internationally or even inter-provincially, comparing absolute costs between studies is impractical [38]. However, it is important to note that some cancer types show a similar trend of increased expense with later stage disease [39–41], while others do not [42, 43]. In addition, the contributing factors (e.g., hospitalization, medication, etc.) can differ significantly between tumor sites [39].

The only nearly comparable study of resource utilization in Ontario was that reported by Mittmann et al. [31] in which incident cases of BC diagnosed from 2005-2009 had an average 2-year cost of $29,938, $46,893, $65,369, and $66,627 in stage I, II, III and IV, respectively. This is in contrast to our more contemporary and HER2+ -subtyped cohort in which we report an average annual cost of $54,852 and $159,348 in patients with stage I–III and stage IV disease, respectively. Pharmaceuticals were the largest cost contributor in our cohort, and increased use of targeted therapies for HER2+ BC over time likely explains the higher costs in our cohort compared to Mittmann et al. [31]. We encourage the use of our more recent, real-world data points (e.g., neoadjuvant treatment rates and costs associated with metastatic disease) in future cost-effectiveness analyses of novel treatment interventions (e.g., trastuzumab emtansine).

## Limitations

Limitations of our study include those inherent to administrative claims database analyses, such as missing clinical variables (e.g., ethnicity, menopausal status, genetic test results, and recurrence or progression). Notably, a lack of recurrence data restricted our ability to identify costs related to distant recurrence from the stage I–III cohort and apply them to the stage IV cohort. Therefore, costs reported for the stage I-III cohort may be inflated. With a province-wide, population-based sample, potential biases were limited, but sources could include the exclusion of patients without histologic subtype (*n* = 3914) or tumor staging (*n* = 13) information, or the lack of prescription drug data for patients under 65 years of age.

Because only publicly funded services were captured in the databases utilized, a true total cost of care cannot be calculated (i.e., out of pocket expenses). In addition, cost versus matched controls without BC was not assessed; thus, the total costs reported for these HER2+ BC patients represent measurable publicly funded health care costs and not only BC-attributable costs.

## Conclusion

While there were substantially fewer cases of de novo stage IV HER2+ BC compared with earlier stage disease, the resource cost in this cohort was three times higher and not insignificant, with 36.6% living to 5 years [44]. While the personal benefit of optimizing NAT/AT and decreasing the risk of future metastatic disease is implicit for women with early HER2+ BC, our study reflects the potential for a similarly positive cost benefit of such a strategy to a publicly funded health care system. To this end, our cohort shows potential for higher neoadjuvant treatment rates. Understanding real-world costs and treatment patterns in HER2+ disease should assist in the development of optimal care pathways.

## Electronic supplementary material

Below is the link to the electronic supplementary material.

Supplementary material 1 (DOCX 169 kb)

## Data Availability

The dataset from this study is held securely in coded form at the Institute for Clinical Evaluative Sciences (ICES). While data sharing agreements prohibit ICES from making the dataset publicly available, access may be granted to those who meet pre-specified criteria for confidential access, available at www.ices.on.ca/DAS.
