# Supplementary material for: A population-based comparison of treatment, resource utilization, and costs by cancer stage for Ontario patients with HER2-positive breast cancer
Source: Breast Cancer Res Treat. 2020 Oct 22;185(3):807–15. doi: 10.1007/s10549-020-05976-w (PMC7921035; doi:10.1007/s10549-020-05976-w)
Supplement: Supplementary file 1 — Supplementary material 1 (DOCX 169 kb) [file 10549_2020_5976_MOESM1_ESM.docx]

**SUPPLEMENTARY INFORMATION**

**for**

***A population-based comparison of treatment, resource utilization and costs***

***by cancer stage for Ontario patients with HER2-positive breast cancer***

***Breast Cancer Research and Treatment***

Christine Brezden-Masley MD, PhD, FRCPC^1^ | Kelly E. Fathers PhD^2^ | Megan E. Coombes MSc^3^ | Behin Pourmirza MD^2^ | Cloris Xue MSc^2^ | Katarzyna J. Jerzak MD, MSc, FRCPC^4^

^1^Division of Medical Oncology and Hematology, Faculty of Medicine, University of Toronto, Mount Sinai Hospital (Toronto, Ontario, Canada)

^2^Department of Medical Affairs, Hoffmann-La Roche Limited (Mississauga, Ontario, Canada)

^3^Market Access and Pricing Department, Hoffmann-La Roche Limited (Mississauga, Ontario, Canada)

^4^Division of Medical Oncology and Hematology, Faculty of Medicine, University of Toronto, Sunnybrook Odette Cancer Center (Toronto, Ontario, Canada); katarzyna.jerzak@sunnybrook.ca

**Fig. S1** CONSORT diagram of exclusions to arrive at final cohort of female cases of HER2+ breast cancer diagnosed and staged between Apr 1, 2012 and Mar 31, 2016 from the ICES database

Breast cancer diagnosed between Apr 1 2012 and Mar 31 2016 with valid IKN

N=40,929

Exclude: concurrent cancer diagnosis

N=92

Remaining cases

N=40,837

Exclude: previous cancer diagnosis

N=6076

Remaining cases

N=34,761

Exclude: unknown sex

N=0

Remaining cases

N=34,761

Exclude: age <18 or >105

N=252

Remaining cases

N=34,509

Exclude: death date before diagnosis

N=8

Remaining cases

N=34,501

Exclude: malignant lymphomas

N=161

Remaining cases

N=34,340

Exclude: HER2 negative

N=25,524

Remaining cases

N=8,816

Exclude: HER2, ER or PR unknown

N=3,914

Remaining cases

N=4,902

Exclude: AJCC stage unknown

N=13

**Final Study Cohort**

**N=4,889**

Abbreviations: AJCC, American Joint Committee on Cancer; HER2, human epidermal growth factor receptor 2; ER, estrogen receptor; IKN, encrypted ICES key number; PR, progesterone receptor.

**Table S1** List and description of datasets available to ICES

| **Dataset** | **Related Variable** | **Description** |
| --- | --- | --- |
| **Owner: Cancer Care Ontario** | | |
| Cancer Activity Level Reporting (ALR) *2005* | - Systemic therapy - Supportive drugs  - Radiation therapy | Contains patient level activity within the cancer system focused on radiation and systemic therapy services and outpatient oncology clinic visits. |
| **Owner: Ontario Ministry of Health and Long term Care** | | |
| Client Agency Program Enrolment (CAPE) *1999* | - Capitation costs | Registry of patients enrolled in a primary care model. Data elements include program type (family health team, family health organization, family health network, etc.) and patient enrolment status. |
| Home Care Database (HCD), Ontario Home Care Administrative System (OHCAS) *1990* | - Home care services | Captures information on all services provided or coordinated by Ontario Community Care Access Centres, including client data, intake and assessment information, admission and discharge, etc. |
| New Drug Funding Program (NDFP) *1995* | - Medication use including systemic therapy | Administered by Cancer Care Ontario, the NDFP funds new, and often very expensive, cancer drugs. |
| Ontario Cancer Registry (OCR) *1964* | - Cancer diagnosis  - Tumour characteristics (molecular) | Contains the diagnosis code for invasive cancer (International Classification of Diseases version 10) and the date of diagnosis for all residents of Ontario. |
| Ontario Drug Benefit (ODB) claims *1990* | - Medication use including systemic therapy | Contains claims for oral prescription drugs covered under the ODB program. Primarily includes drug claims for individuals 65 years of age and older, but also coverage under special ODB programs. |
| Ontario Health Insurance Plan (OHIP) claims database *1991* | - Outpatient physician visits - Laboratory services - Non-physician services  - Physician services (including costs related to breast reconstruction) | Contains claims paid by OHIP, the universal, single-payer provincial health insurance plan, for services provided by all eligible health care providers, including physicians (primary and specialist), groups, and laboratories. |
| Registered Persons Database (RPDB) *1991* | - Health service subscriber data | Contains birth and death dates, age, sex, and date of last contact with health care services in Ontario. |
| **Owner: Canadian Institute for Health Information** | | |
| Continuing Care Reporting System (CCRS) *1996* | - Complex continuing care (CCC) - Long-term care (LTC) | Contains information about residents receiving facility-based continuing care services. Range of services includes CCC, extended or chronic care, and residential care providing nursing services (that is, long-term care). |
| Discharge Abstract Database (DAD) *1988* | - Inpatient hospitalizations | Contains demographic, clinical, and administrative data for inpatient hospital admissions (patient separations). |
| National Ambulatory Care Reporting System (NACRS) *2000/2003* | - Ambulatory emergency department visit, dialysis clinic visits, and cancer clinic visits | Contains data from hospital- and community-based ambulatory care services, including same day surgery, outpatient clinics, and emergency departments. |
| National Rehabilitation Reporting System (NRS) *2000* | - Rehabilitation admissions | Contains client data from adult inpatient rehabilitation facilities, such as administrative data (referral, admission, and discharge) and health and functional characteristics. |
| Ontario Mental Health Reporting System (OMHRS) *2005* | - Mental health admissions | Contains data on patients in adult designated inpatient mental health beds in acute and psychiatric facilities. Data elements include admission and discharge dates, diagnoses, service utilization, etc. |

Reference: ICES Data Dictionary [Internet]. 2019. Available from: https://datadictionary.ices.on.ca/Applications/DataDictionary/Default.aspx

| **Table S2** Treatments and wait times among patients with stage I-III HER2+ breast cancer (Ontario, 2012–2017) | | | | | | | |
| --- | --- | --- | --- | --- | --- | --- | --- |
|  | **Variable** | **Value** |  | **Stage I-III with surgery (n=4,372)** |  | **Stage I-III without surgery (n=435)** |  |
|  | **Surgery** |  |  |  |  |  |  |
|  | No. of surgeries within 1 year of dx | Mean (SD) |  | 1.17 (0.40) |  | NA |  |
|  | Days between dx and surgery | Mean (SD) |  | 66 (67) |  | NA |  |
|  |  | Median (IQR) |  | 38 (25–70) |  | NA |  |
|  | **Systemic Therapy** |  |  |  |  |  |  |
|  | Patients who received | N (%) |  |  |  | 128 (78.5) |  |
|  | Days between dx and first tx | Mean (SD) |  |  |  | 123 (210) |  |
|  |  | Median (IQR) |  |  |  | 47 (29–100) |  |
|  | **Radiation Therapy** |  |  |  |  |  |  |
|  | Patients who received | No. (%) |  | 3,450 (78.9) |  | 71 (43.6) |  |
|  | Days between dx and first tx | Mean (SD) |  | 206 (96) |  | 359 (313) |  |
|  |  | Median (IQR) |  | 201 (172–231) |  | 224 (136–537) |  |
| Abbreviations: dx, diagnosis; IQR, interquartile range; NA, not applicable; tx, treatment | | | | | | | |

| **Table S3** Treatments and wait times among patients with stage IV HER2+ breast cancer (Ontario, 2012–2017) | | | | | | | |
| --- | --- | --- | --- | --- | --- | --- | --- |
|  | **Variable** | **Value** |  | **Stage IV**  **with surgery (n=82)** |  | **Stage IV without surgery (n=272)** |  |
|  | **Surgery** |  |  |  |  |  |  |
|  | No. of surgeries within 1 year of dx | Mean (SD) |  | 1.01 (0.11) |  | NA |  |
|  | Days between dx and surgery | Mean (SD) |  | 138 (105) |  | NA |  |
|  |  | Median (IQR) |  | 168 (34–234) |  | NA |  |
|  | Patients who received surgery only | N (%) |  | 3 (3.7)*^a^* |  | NA |  |
|  | **Systemic Therapy** |  |  |  |  |  |  |
|  | Patients who received systemic tx | N (%) |  | 78 (95.1)*^a^*  45 (54.9)*^b^* |  | 235 (86.4) |  |
|  | Days between dx and first tx | Mean (SD) |  | 30 (20)*^b^* |  | 50 (66) |  |
|  |  | Median (IQR) |  | 27 (18–41)*^b^* |  | 36 (22–51) |  |
|  | Days between first tx and surgery | Mean (SD) |  | 196 (47)*^b^* |  | NA |  |
|  |  | Median (IQR) |  | 186 (168–213)*^b^* |  | NA |  |
|  | **Radiation Therapy** |  |  |  |  |  |  |
|  | Patients who received | N (%) |  | 53 (64.6) |  | 160 (58.8) |  |
|  | Days between dx and first tx | Mean (SD) |  | 263 (220) |  | 229 (295) |  |
|  |  | Median (IQR) |  | 247 (148–283) |  | 59 (18–399) |  |
| *^a^*mid-point of suppressed data range, n=$\pm$2.  *^b^*in those receiving systemic tx before surgery.  Abbreviations: dx, diagnosis; IQR, interquartile range; tx, treatment. | | | | | | | |

| **Table S4** Number of visits, length of stay, and cost (CAD) per person per year (mean ± SD) for each health care resource by stage in HER2+ breast cancer (Ontario, 2012-2017) | | | | | | | | | | |
| --- | --- | --- | --- | --- | --- | --- | --- | --- | --- | --- |
|  |  |  | **Full sub cohort** | | |  | **Population utilizing** | | |  |
|  | **Resource** |  | **Stage I-III**  **(n=4,535)** |  | **Stage IV**  **(n=354)** |  | **Stage I-III** |  | **Stage IV** |  |
|  | **Professional (OHIP)** | | |  |  |  | **n=4,532 (100%)*^a^*** |  | **n=354 (100%)** |  |
|  | Visits (no.) |  | 47.5 ± 33.6 |  | 135.9 ± 161.1 |  | 47.5 ± 33.6 |  | 135.9 ± 161.1 |  |
|  | Cost |  | $5,922 ± 4,184 |  | $15,687 ± 18,274 |  | $5,923 ± 4,183 |  | $15,687 ± 18,274 |  |
|  | **Lab (OHIP)** |  |  |  |  |  | **n=4,278 (94%)** |  | **n=265 (75%)** |  |
|  | Visits (no.) |  | 17.7 ± 19.8 |  | 17.8 ± 46.0 |  | 18.7 ± 19.9 |  | 23.7 ± 51.9 |  |
|  | Cost |  | $145 ± 136 |  | $180 ± 411 |  | $154 ± 135 |  | $240 ± 459 |  |
|  | **Inpatient (Hosp.)** | | |  |  |  | **n=2,513 (55%)** |  | **n=264 (75%)** |  |
|  | Visits (no.) |  | 0.5 ± 0.9 |  | 3.8 ± 20.2 |  | 0.8 ± 1.0 |  | 5.1 ± 23.3 |  |
|  | LOS (days) |  | 2.5 ± 10.8 |  | 34.3 ± 84.0 |  | 4.4 ± 14.2 |  | 46.0 ± 94.6 |  |
|  | Cost |  | $3,769 ± 13,183 |  | $43,793 ± 117,213 |  | $6,802 ± 17,118 |  | $58,723 ± 132,515 |  |
|  | **Inpatient (Rehab.)** | | |  |  |  | **n=60 (1%)** |  | **n=14 (4%)** |  |
|  | Visits (no.) |  | 0.0 ± 0.1 |  | 0.0 ± 0.2 |  | 0.4 ± 0.3 |  | 0.8 ± 0.6 |  |
|  | LOS (days) |  | 0.2 ± 2.3 |  | 0.7 ± 4.2 |  | 11.8 ± 16.2 |  | 17.1 ± 13.5 |  |
|  | Cost |  | $112 ± 1,278 |  | $464 ± 2,712 |  | $8,472 ± 7,308 |  | $11,730 ± 7,582 |  |
|  | **Inpatient (MH)** |  |  |  |  |  | **n=14 (0%)*^a^*** |  | **n=3 (0%)*^a^*** |  |
|  | Visits (no.) |  | 0.0 ± 0.1 |  | 0.0 ± 0.2 |  | 0.6 ± 0.5 |  | … |  |
|  | LOS (days) |  | 0.1 ± 3.5 |  | 0.3 ± 6.1 |  | 22.9 ± 55.8 |  | … |  |
|  | Cost |  | $61 ± 2,689 |  | $190 ± 3,567 |  | $17,188 ± 43,265 |  | … |  |
|  | **Same Day Surgery** | | |  |  |  | **n=3,773 (83%)** |  | **n=112 (32%)** |  |
|  | Visits (no.) |  | 0.6 ± 0.6 |  | 0.3 ± 1.0 |  | 0.7 ± 0.5 |  | 0.9 ± 1.6 |  |
|  | Cost |  | $1,468 ± 1,329 |  | $483 ± 1,330 |  | $1,764 ± 1,265 |  | $1,527 ± 2,005 |  |
|  | **Hospital Outpatient** | | |  |  |  | **n=4,506 (99%)** |  | **n=351 (100%)*^a^*** |  |
|  | Visits (no.) |  | 6.5 ± 6.1 |  | 16.7 ± 32.8 |  | 6.6 ± 6.1 |  | 16.8 ± 32.8 |  |
|  | Cost |  | $2,238 ± 2,088 |  | $5,876 ± 11,577 |  | $2,252 ± 2,087 |  | $5,910 ± 11,602 |  |
|  | **Home Care** |  |  |  |  |  | **n=3965 (87%)** |  | **n=298 (84%)** |  |
|  | Cost |  | $1,499 ± 3,124 |  | $6,146 ± 10,963 |  | $1,714 ± 3,285 |  | $7,301 ± 11,592 |  |
|  | **Amb. Cancer** |  |  |  |  |  | **n=4,257 (94%)** |  | **n=319 (90%)** |  |
|  | Cost |  | $19,146 ± 12,543 |  | $28,376 ± 17,925 |  | $20,397 ± 11,921 |  | $31,490 ± 16,073 |  |
|  | **Amb. Emergency** | | |  |  |  | **n=3,200 (71%)** |  | **n=294 (83%)** |  |
|  | Cost |  | $343 ± 626 |  | $1,612 ± 3,741 |  | $487 ± 697 |  | $1,941 ± 4,027 |  |
|  | **Amb. Dialysis** |  |  |  |  |  | **n=13 (0%)** |  | **n=0** |  |
|  | Cost |  | $122 ± 3,110 |  | $0 ± 0 |  | $42,714 ± 41,019 |  | $0.00 ± 0.00 |  |
|  | **Drug (NDFP)** |  |  |  |  |  | **n=3,794 (84%)** |  | **n=283 (80%)** |  |
|  | Cost |  | $17,495 ± 14,796 |  | $49,691 ± 41,116 |  | $20,912 ± 13,792 |  | $62,158 ± 36,586 |  |
|  | **Drug (ODB)** |  |  |  |  |  | **n=3,591 (79%)** |  | **n=269 (76%)** |  |
|  | Cost |  | $1,958 ± 3,109 |  | $2,009 ± 4,164 |  | $2,473 ± 3,307 |  | $2,644 ± 4,599 |  |
|  | **CCC** |  |  |  |  |  | **n=76 (2%)** |  | **n=42 (12%)** |  |
|  | Cost |  | $212 ± 2,735 |  | $4,319 ± 20,548 |  | $12,648 ± 17,110 |  | $36,402 ± 49,385 |  |
|  | **Long Term Care** | | |  |  |  | **n=64 (1%)** |  | **n=7 (2%)** |  |
|  | Visits (no.) |  | 0.1 ± 0.6 |  | 0.1 ± 0.7 |  | 4.3 ± 3.0 |  | 4.0 ± 3.1 |  |
|  | LOS (days) |  | 2.8 ± 29.0 |  | 3.7 ± 32.2 |  | 198.4 ± 144.7 |  | 187.9 ± 143.8 |  |
|  | Cost |  | $361 ± 3,795 |  | $522 ± 4,594 |  | $25,545 ± 19,564 |  | $26,397 ± 21,085 |  |
| *^a^*mid-point of suppressed data range, n=$\pm$2.  Abbreviations: Amb., ambulatory; CCC, Complex Continuing Care; Hosp., hospital; LOS, length of stay; LTC, MH, Mental Health ; NDFP, New Drug Funding Program; ODB, Ontario Drug Benefit; OHIP, Ontario Health Insurance Plan; Rehab., rehabilitation; SDS, Same Day Surgery; TNBC, triple negative breast cancer. | | | | | | | | | | |
